# Sociopsychological determinants of functional foods consumption in China: based on the theory of the planned behavior expansion model

**DOI:** 10.3389/fnut.2025.1624390

**Published:** 2025-08-01

**Authors:** Huimin Jia, Shuang Liu

**Affiliations:** Shanxi Institute for Functional Food, Shanxi Agriculture University, Taiyuan, Shanxi, China

**Keywords:** functional foods, health consciousness, trust, theory of planned behavior, purchase intention

## Abstract

This study investigates the determinants of Chinese consumers’ purchase intentions and behavior toward functional foods, incorporating health consciousness and trust into the theory of planned behavior (TPB) as a conceptual framework. An online quota-sample survey was conducted on 1,011 Chinese consumers. Structural equation modeling was used to test the proposed hypotheses. Three main findings were drawn based on the analysis: (1) Health consciousness significantly improved consumers’ attitudes towards functional foods (*β* = 0.921, *p* < 0.001); (2) Consumption intention was predominantly predicted by attitude (*β* = 0.751, *p* < 0.001), followed by perceived behavioral control (*β* = 0.148, *p* < 0.05) and trust (*β* = 0.115, *p* < 0.001). (3) Contrary to theoretical expectations, the positive influence of subjective norm (*β* = 0.222, *p* > 0.05) on purchase intention is not significant, and the purchase intention did not significantly influence behavior. Notably, perceived behavioral control (*β* = 0.841, *p* < 0.001) emerged as the strongest direct predictor of consumption behavior. This research empirically validates the explanatory power of the expanded Theory of Planned Behavior (TPB) in the context of functional food consumption. The results establish a more detailed conceptual framework for understanding consumption mechanisms, while providing evidence-based strategies for market development and public health interventions.

## 1 Introduction

National public health policies are placing greater emphasis on fostering healthier dietary practices. The prevalence of diet-related non-communicable chronic diseases (NCDs) in China has resulted in a heavy epidemic burden and huge economic losses, with serious implications for the well-being of its population (1). With the population aging, rapid urbanization, and continuous changes in lifestyle, diet-related health issues are becoming increasingly prevalent (2, 3). Functional foods are recognized as an effective way to address diet-related health problems.

Functional foods are defined as products “that are demonstrated to affect beneficially one or more target functions in the body, beyond adequate nutritional effects, in a way that is relevant to either improved state of health and well-being and/or reduction of risk of disease” (4). In recent years, functional food sales have expanded rapidly in industrialized countries, primarily in the United States, Europe, Japan, and Canada (5). Meanwhile, China, as an emerging market, has also witnessed significant growth. In 2017, the National Development and Reform Commission and the Ministry of Industry and Information Technology of the People’s Republic of China jointly issued the “Guiding Opinions on Promoting the Healthy Development of the Food Industry” to encourage and support the development of functional foods (6). Since then, functional food research in China has emerged as a hotspot and cutting-edge field (7). Public’s interest in healthy food and government initiatives have driven food manufacturers to actively launch a variety of new functional foods aimed at improving the health of the Chinese population, further promoting sustained growth in the Chinese functional food market. However, issues related to functional foods consumption occurred intensively. In China, functional foods lack a clear legal definition and are regulated under the category of general foods, which can cause confusion in the market and among consumers. In 2023, the State Administration for Market Regulation organized special operations to monitor advertisements affecting people’s livelihoods. They investigated and addressed 47,600 cases of false or illegal advertising (8). Consumers have shown different attitudes towards functional foods, including scepticism about their efficacy and criticism about their cost-effectiveness. Practitioners and researchers have recognized the importance of consumer awareness and acceptance for the successful development and marketing of functional foods (9). Therefore, it is necessary to investigate the factors influencing consumer’ purchase intentions and behavior regarding functional foods.

Empirical studies have examined several core factors that influence the consumption of functional foods. Perceptions of food health play a crucial role in the decision to consume a functional food (10, 11). Key determinants of these perceptions include cognitive factors such as perceived health (12); knowledge of nutrients (13), label/nutritional information (14), and trust in product safety and efficacy (14, 15). Other factors that influence consumer perceptions include product attributes, such as ingredient information (e.g., proteins, sodium and carbohydrates) (16), convenience (17), price (15), taste (18, 19), and evidence-based health claims (20). Moreover, cultural values influence the acceptance of functional foods, with differences in intention and actual consumption across countries (16). Previous research has shown that Chinese consumers exhibit a higher propensity to purchase functional foods than Western European consumers (21). However, Mirosa and Mangan-Walker note that while Chinese consumers attach significant importance to their mobility health, the accessibility and personal use of functional foods still need further investigation (22).

The Theory of Planned Behavior (TPB) has been widely employed as the primary theoretical framework for interpreting intentions and behavior regarding the consumption of functional foods. However, the cross-cultural validity of the model needs to be verified in the context of China’s unique dietary traditions and health concepts (23). O’Connor used the TPB to examine the willingness of Australian non-users to try vitamin supplements and functional foods (24). While this study examined the inhibitory effect of risk dread on consumption intentions, it did not investigate whether frequent safety concerns in the Chinese market exacerbate the gap between intention and behavior. Nystrand and Olsen investigated the antecedents of Norwegian consumers’ attitudes and intentions to consume functional foods using an extended TPB model (25). While the Norwegian study revealed that expanding the range of variables increased the model’s explanatory power by including hedonic and utilitarian eating values, and self-efficacy, the Western sample was unable to account for the profound influence of Chinese cultural tradition on health perceptions, particularly the idea that “medicine and food share a common origin” (26). Lim and An applied the TPB to predict Korean consumers’ purchase intentions for well-being food, namely Yak-sun (27). Pienwisetkaew et al. examined the significant relationships among factors influencing the purchase intentions for functional non-dairy milk using the TPB framework (28). Given the high diversity of China’s functional food market, targeted research remains essential. Studying Chinese consumers could help address the theoretical gap relating to cultural differences and provide a robust foundation for local enterprises to formulate product development and marketing strategies.

Therefore, this study aims to use an expanded theory of planned behavior (TPB) framework integrated with health consciousness and trust to comprehensively explore Chinese people’s intentions and behaviors regarding functional food consumption. The findings are expected to make a significant theoretical contribution and provide practical insights into developing effective marketing strategies and health communication for Chinese consumers.

## 2 Conceptual model

The TPB used in this study is a sociopsychological theory proposed by Icek Ajzen, which can predict and explain human behavior in specific situations (29). According to the TPB, attitude, subjective norm and perceived behavioral control (PBC) predict behavioral intention, the latter of which is directly related to behavioral performance (30). The theory of planned behavior has been extensively applied in various domains both domestically and internationally, including consumer and health behaviors. Numerous studies on health behaviors have demonstrated the substantial explanatory and predictive power of this theory (31), including physical activity (32), vaccination (33), fruit consumption (34), whole-grain food consumption (35), and fast food alternatives (36).

Many researchers have found that the parsimonious structure of the TPB model makes it impossible to explain the complex motives behind some behavioral choices. Scholars in diverse research fields have improved and expanded the TPB by considering the characteristics of the research objects to make it more applicable to a variety of behavioral decision-making scenarios (30). Liao et al. examined the determinants of takeaway waste separation intention among Chinese urban residents by adding three factors to the TPB model (37): environmental concerns, facilities, and time pressure. Varah et al. incorporated the willingness to pay price premiums and environmental concerns into the TPB to examine young consumers’ intentions towards green products (38). Sun and Moon extended the TPB by using price fairness, healthiness, eco-friendliness, and ease of use as the four antecedents of attitude to analyze the drivers of bee propolis products (39). Therefore, this study uses TPB to measure consumers’ intentions and behavior towards functional foods, and expands it to include additional dimensions relevant to this specific topic.

**Figure 1:**
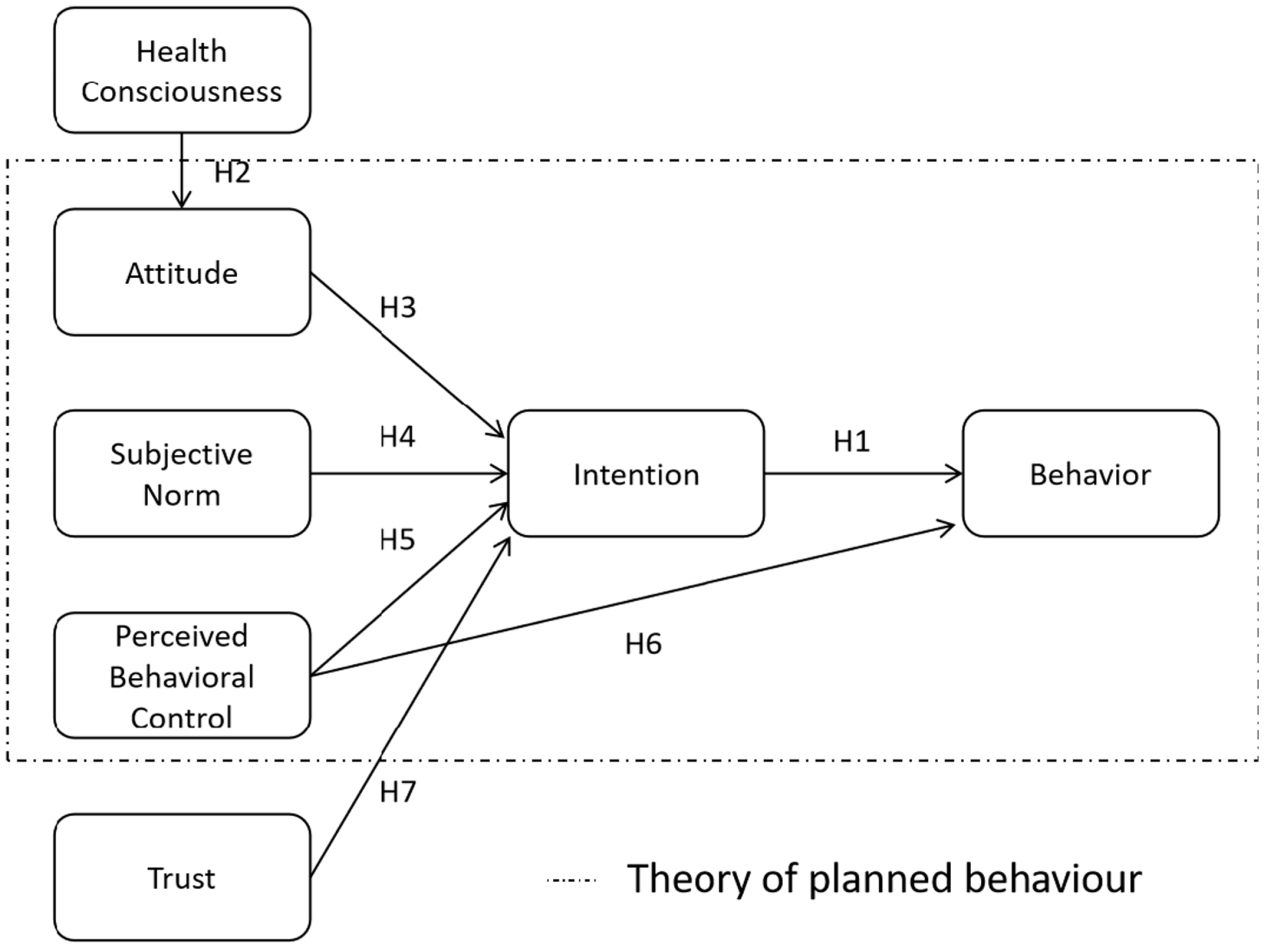
Framework of the expanded theory of planned behavior model.

As discussed, the TPB comprises constructs such as attitude, subjective norm, and PBC. In this study, we modified the TPB by including health consciousness as an antecedent of attitude and trust as an antecedent of intention (Figure 1).

### 2.1 Intention and consumption behavior

According to Ajzen, TPB suggests that intention most directly influences behavioral performance; intention refers to the “tendency to try to perform a behavior” (29), and the greater the intention to perform a behavior, the greater the likelihood that the action will be carried out. A review of the theory of planned behavior and the rational behavior approach suggests that intention explains between 18 and 23% of the variance across a wide range of behaviors (31), particularly health behaviors (40). In healthy dietary practices, researches have demonstrated that intentions exert a positive influence on eating behavior (41, 42).

In studies using the TPB framework, the assessment of attitudinal constructs and behavioral outcomes adheres to the logically prescribed chronological sequence dictated by theoretical postulates (43). Due to difficulties in contacting respondents in the days immediately following the distribution of the questionnaire to assess their functional food consumption behavior, administration of both behavioral and attitudinal measurements was necessary. However, dietary choices remain relatively stable over short periods of time, so the simultaneous or delayed measurement of consumption behavior does not yield substantial differences (44). Therefore, data derived from retrospective behavioral surveys can be used as a valid proxy for predicting future behavior (45). In this study, a retrospective behavioral survey was employed to measure behavioral constructs. Thus, the following hypothesis is proposed:

*H1*: Consumer intention significantly impacts their purchase behavior toward functional foods.

### 2.2 Health consciousness

Health consciousness refers to a person’s willingness to take action for their health (46). Empirical evidence consistently shows that health-conscious consumers prioritize wellness-oriented products and have a stronger intention to purchase health-promoting goods and services (28). Specifically regarding functional foods: Chen identified the health-oriented consumers hold significantly more positive attitudes towards them (47). Cranfield et al. found that perceived health status was associated with attitudes towards functional foods among women, and individuals who were aware of the strong link between the nutritional benefits of functional foods and their personal health were more likely to consume such products (5). This finding implies that consumers’ health consciousness positively influences their attitudes towards functional foods. Thus, the following hypothesis is proposed:

*H2*: Health consciousness significantly impacts consumers’ attitudes towards purchasing functional foods.

### 2.3 Attitude

In the TPB framework, attitude is recognized as a determinant of intention. Attitude refers to one’s positive or negative perceptions of performing a particular action (29). It is often cited as the most robust predictor of the intention to engage in healthy eating practices (12, 24, 48–50). Carrillo et al. demonstrated that consumers’ attitudes can serve as a reliable predictor of spending on functional foods (12). Patch et al. also revealed that attitudes were the primary determinant of Australian consumers’ intention to consume foods fortified with omega-3 fatty acids (51). Our study suggests that individuals with positive attitudes towards functional foods are more likely to incorporate them into their diets. Thus, the following hypothesis is proposed:

*H3*: Attitude significantly impacts consumers’ purchase intention towards functional foods.

### 2.4 Subjective norm

Subjective norms refer to an individual’s perception of the social support they receive from important significant others, such as family members, friends, or colleagues, when deciding whether to perform a specific behavior (29). Previous studies confirmed their predictive validity in health contexts. For example, O’Connor demonstrated a significant influence on the adoption of health products (24). Patch et al. reported that family and friends play a crucial role in introducing new foods to individuals, while dietitians were regarded as reliable information sources. In contrast, food companies and scientists were found to be the least trusted among the information providers (52). China exhibits an acquaintance-based cultural characteristic, where individual behaviors are significantly shaped by social influences (14). Thus, the following hypothesis is proposed:

*H4*: Subjective norms significantly impact consumers’ purchase intention towards functional foods.

### 2.5 Perceived behavioral control

Perceived behavioral control is defined as an individual’s subjective assessment of their ability to control a particular behavior, including how easy or difficult they perceive it to be (29). This construct exerts a dual influence, acting directly on behavioral enactment and indirectly via behavioral intentions. Empirical evidence demonstrates its critical role in food-related decision-making processes (29). For instance, reduced perceived difficulty increases the likelihood of online purchasing (53). Studies focusing on functional foods emphasize the importance of PBC factors. For example, Marimuthu found that perceived convenience was the main factor influencing young mothers’ decisions to administer herbal food supplements to their children (54). Nolan-Clark et al. revealed that some consumers had challenges interpreting dairy nutrition labels due to limited time and insufficient skills (55). Lalor et al. further reported that price was one of the main influences on the purchase of healthy foods (56). In our study, we follow the original hypothesis of the TPB model, and posit that consumers are more likely to consume functional foods when they have the requisite conditions to do so. Thus, the following hypotheses are proposed:

*H5*: Perceived behavioral control significantly impacts consumers’ purchase intention towards functional foods.

*H6*: Perceived behavioral control significantly impacts consumers’ purchase behavior towards functional foods.

### 2.6 Trust

Trust refers to “a psychological state characterized by the willingness to accept vulnerability based on positive expectations of other’s intentions or actions” (57). Functional foods are considered to have health-promoting properties in addition to providing adequate nutrition (4). However, crucially, consumers require confidence in the tangible health benefits of regular consumption before they will adopt them (58). This study considers three dimensions of trust: personalized, institutional, and public trust in technology (59). “Personalized trust” refers to consumers’ confidence in food products from short supply chains and small-scale production systems. This trust is built and sustained through consumers’ in-depth knowledge of food origins and production processes. In contrast, large-scale supply systems predominantly rely on “institutionalized trust” (60), which is defined as the acceptance and perception that the system is benevolent, competent, reliable, and accountable (61). Public trust in technology manifests as confidence in food processing methods and associated health benefits (16). Empirical evidence has shown that trust is critical to consumers’ food purchase decisions (58, 59) Trust in technology has been shown to influence both perceived risk and perceived benefit (62); while trust in food organizations and systems affects consumer preferences and product acceptance (63, 64). These findings suggest that trust positively influences consumers’ intentions towards functional foods. Thus, the following hypothesis is proposed:

*H7*: Trust significantly impacts consumers’ purchase intention towards functional foods.

## 3 Materials and methods

### 3.1 Data collection and sample

This study adopts a quantitative approach utilizing primary data gathered via an online survey. The data were analyzed using structural equation modeling (SEM). In order to obtain stable parameter estimates and standard errors, the sample size should be sufficiently large. According to research by Schumacker and Lomax, samples of between 250 and 500 are generally sufficient (65). Given China’s large population and vast size, the sample size for this study was increased to 1,000. After excluding responses from participants under the age of 18, 1,011 valid data were collected from Chinese adult consumers during the empirical study.

The participants were recruited and randomly selected from an external data collection agency. Gender, age, and income quotas reflecting the composition of the Chinese population were used to ensure a national representative sample. The sample included 581 male participants (57.5%); 25.5% were aged between 40 and 49 years, and 23.7% were aged between 18 and 20 years. The majority of respondents were well educated, with 420 (41.5%) having completed high school or vocational senior high school, and 330 (32.7%) having a college diploma or above. Table 1 summarizes some of the sociodemographic characteristics of the sample. Due to the retrospective, anonymous nature of this study and the fact that no personally sensitive information was collected, our Institutional Review Board waived the need for informed consent.

**Table 1:** Sociodemographic characteristics of the study population (*n* = 1,011).

Variables	Categories	Frequency	Percent (%)
Gender	Male	581	57.5
	Female	430	42.5
Age	18–29 years old	240	23.7
	30–39 years old	215	21.3
	40–59 years old	300	29.7
	60 years old or above	256	25.3
Marital status	Married	783	77.4
	Unmarried	210	20.8
	Divorced	7	0.7
	Widowed	11	1.1
Education status	Middle school or below	261	25.8
	High school or vocational senior high school	420	41.5
	College diploma or above	330	32.7
Monthly per capita household income	RMB 2,000 or below	31	3.1
	RMB 2,001–3,000	449	44.4
	RMB 3,001–4,000	227	22.5
	RMB 4,001–5,000	202	20.0
	5,000 or above	102	10.1

### 3.2 Questionnaire development

The questionnaire commenced with a brief description of the term “functional foods” used in this study. The term was defined in this study as a food product that, in addition to its usual nutritional value, has a specific effect on maintaining or promoting health or reducing the risk of disease (10). The questionnaire comprises two parts. The first part contained sociodemographic information about the respondents, including age, gender, education level, occupation, and monthly household per capita income. The second part was the expanded theory of planned behavior scale, which measured the variables of the TPB model according to Ajzen (66) outline for constructing a standardized TPB questionnaire with reference to the items commonly used in previous studies to formulate the items for the two additional variables.

### 3.3 Measurement of expanded TPB variables

Consumer purchasing behavior toward functional foods was evaluated using three items measuring consumption frequency (BEH1), duration (BEH2) and percentage of dietary intake (BEH3). Each item used a distinct 5-point ordinal scale (66, 67). Purchase intention was captured through four items addressing intention, expectation, trial and planning to consume regularly (INT1–INT4). Attitudes were evaluated using four items addressing perceived benefits, pleasantness, value and the goodness of consumption (ATT1–ATT4). Subjective norms were measured using four items reflecting the perceived beliefs, expectations, endorsement and understanding of acquaintances (SN1–SN4). Perceived behavioral control was measured using three items concerning consumption control, including one reverse-scored item (PBC1–PBC3). Health consciousness was assessed using three items concerning self-perception, willingness to sacrifice and essential knowledge (HC1–HC3). These items were selected from the 11 items of the Health Consciousness Scale developed by Ophuis (46). Trust was measured using three items concerning the nutritional adequacy of functional foods, the regulatory authorities and the producer integrity (TR1–TR3) (16). All constructs except behavior used a five-point Likert scale ranging from 1 (strongly disagree) to 5 (strongly agree). Appendix shows the details of variable constructs and items.

### 3.4 Data analysis

Initial analyses using SPSS 25.0 explored data and confirmed the normality of distributions. Subsequently, a two-stage procedure in AMOS 24.0 was used for confirmatory factor analyses (CFA) and SEM. CFA verified the validity of the measurement model (68), while SEM examined the hypothesized causal pathways (69). The internal consistency of each scale was assessed using Cronbach’s alpha coefficient. Convergent validity of the measures were evaluated using composite reliability (CR) and average variance extracted (AVE). The structural model testing hypotheses about functional food purchase behavior was assessed using multiple goodness-of-fit indices: *χ*^2^/df < 3, RMSEA< 0.05, and GFI, AGFI, CFI, and IFI and TLI all greater than 0.9 (69, 70). Statistical significance was determined at *α* = 0.05 for all path coefficients.

## 4 Results

### 4.1 The reliability and validity test

The results of the standardized factor loadings, Cronbach’s alpha, C. R., and AVE values, which reflect the reliability and validity of the scale, are shown in Table 2. The Cronbach’s alpha values for the seven variables ranged from 0.628 to 0.789, all of which were close to or above the acceptable threshold of 0.7, indicating good internal consistency of the scale. AVE is a key metric used to evaluate convergent validity. It is calculated as the mean of the squared factor loadings. While factor loadings ideally exceed 0.7, empirical studies often achieve a value of at least 0.6. Therefore, an acceptability threshold of 0.36 for AVE has been established (71). In this study, AVE values ranged from 0.354 to 0.556, all of which met or approached this benchmark. Similarly, CR evaluates internal consistency by aggregating indicator reliability. Higher CR values indicate stronger indicator coherence, with established thresholds designating the following: CR values of at least 0.70 indicate excellent reliability (72), values between 0.60 and 0.69 are considered acceptable for exploratory studies (73), and values below 0.60 necessitate revising the scale to ensure adequate measurement. Contemporary practice emphasizes the contextual interpretation of these benchmarks (74). Our questionnaire produced CR values ranging from 0.630 to 0.790, satisfying the minimum criterion of 0.60. Together, the Cronbach’s alpha, AVE and CR results confirm that the measurement model has adequate convergent validity and internal consistency. The questionnaire data in this study successfully passed the reliability and validity tests, validating its suitability for subsequent analytical procedures.

**Table 2:** Reliability and validity analyses results.

Constructs	Factors	Standardized factor loading	C. R.	AVE	Cronbach’s alpha
Attitude	ATT1	0.659	0.770	0.456	0.770
	ATT2	0.694			
	ATT3	0.675			
	ATT4	0.673			
Subjective norms	SN1	0.597	0.703	0.373	0.699
	SN2	0.646			
	SN3	0.640			
	SN4	0.554			
Perceived behavioral control	PBC1	0.660	0.653	0.389	0.639
	PBC2	0.502			
	PBC3	0.693			
Health consciousness	HC1	0.650	0.630	0.362	0.628
	HC2	0.574			
	HC3	0.579			
Trust	TR1	0.768	0.790	0.556	0.789
	TR2	0.706			
	TR3	0.762			
Intention	INT1	0.585	0.686	0.354	0.684
	INT2	0.605			
	INT3	0.619			
	INT4	0.569			
Behavior	BEH1	0.722	0.783	0.547	0.770
	BEH2	0.803			
	BEH3	0.690			

### 4.2 Measuring model fit

Seven indicators were selected to test the fit of the model for the aforementioned research hypotheses using AMOS software. As can be seen in Table 3, the value of *χ*^2^/df is 2.796 (< 3), which is ideally fitted; the RMSEA is 0.042 (< 0.05), which is ideally fitted; the GFI, AGFI, CFI, and IFI, TLI are all greater than 0.9, and the results are therefore well fitted. Overall, the seven indicators fulfill the corresponding reference standard, and the model demonstrates a good fit.

**Table 3:** Results of the structural equation model fit test.

Indices	*χ*^2^/df	RMSEA	GFI	AGFI	CFI	IFI	TLI
Observed value	2.796	0.042	0.946	0.930	0.945	0.945	0.934
Recommended value	< 3	< 0.05	> 0.90	> 0.90	> 0.90	> 0.90	> 0.90

### 4.3 Testing the hypothesis

Table 4 summarizes the results of the structural equation modeling analysis. A significant positive correlation was found between health consciousness and consumer attitude, as evidenced by a standardized path coefficient of 0.921, which was statistically significant at the 0.05% level. Attitude, perceived behavioral control, and trust were all positively correlated with the intention to purchase functional foods. The respective standardized path coefficients were 0.751, 0.148, and 0.115, respectively, all of which were significant at the 0.05% level. Attitude was found to be the strongest predictor of the intention to purchase functional foods. Perceived behavioral control was also positively related to consumption behavior, as indicated by a standardized path coefficient of 0.841, which was statistically significant at the 0.05% level. However, subjective norms were not significantly positively related to the purchase of functional foods, nor was intention significantly positively related to consumption behavior. These findings support hypotheses H2, H3, H5, H6, and H7, which suggest positive correlations between health consciousness and attitude, and between attitude, perceived behavioral control and trust, and intention. There was also a significant positive correlation between perceived behavioral control and consumption behavior. However, hypothesis H4 was not supported, indicating that the positive correlation between subjective norms and the intention to purchase functional foods is not significant. Similarly, hypothesis H1 was not supported, indicating that there is no significant positive correlation between intention and consumption behavior.

**Table 4:** Structural equation model parameter estimation.

Hypothesis	Path	Estimate	S. E.	C. R.	Std *β*	*t*-values	Hypothesis testing
H1	INT → BEH	0.003	0.088	0.034	0.002	0.973	Not supported
H3	ATT → INT	0.626	0.090	6.978	0.751	0.000	Supported
H4	SN → INT	0.099	0.081	1.221	0.118	0.222	Not supported
H5	PBC → INT	0.130	0.043	3.040	0.148	0.002	Supported
H6	PBC → BEH	1.127	0.102	11.096	0.841	0.000	Supported
H2	HC → ATT	1.793	0.167	10.764	0.921	0.000	Supported
H7	TR → INT	0.059	0.017	3.524	0.115	0.000	Supported

## 5 Discussion and implications

### 5.1 Discussion

This study aims to explore the decision-making influences on Chinese consumers’ consumption of functional foods by introducing health consciousness and trust into the theory of planned behavior, a social psychological theory, to construct a theoretical framework. A questionnaire was administered to 1,011 respondents, and structural equation modeling was used to test the research hypotheses. Most expectations were confirmed, confirming the applicability of the expanded theory of planned behavior in explaining and predicting the intention and consumption behavior towards functional foods among the Chinese population. Finally, the implications, future studies, and limitations of this study are discussed.

We obtained several significant findings. First, health consciousness was found to substantially influence attitudes towards functional foods, as evidenced by a standardized regression coefficient (*β* = 0.921, *p* < 0.001, see Table 4). This robust association, with a value below the multicollinearity threshold of 1, is consistent with previous studies (75). This relationship likely stems from two interrelated mechanisms. The first is health-focused information processing: individuals with heightened health consciousness actively seek nutritional information and critically evaluate health claims, which is consistent with previous research (5). Secondly, value recognition: health-conscious consumers demonstrate a greater appreciation of the nutritional benefits of functional foods, resulting in more favorable attitudes towards them. Furthermore, attitudes were found to be the most important determinant of intention to purchase functional foods (*β* = 0.751, *p* < 0.001, see Table 4). This finding is consistent with previous research (47). Consumers are significantly more likely to purchase functional foods when they fully recognize their nutritional value and are aware of their strong associations with health (5). Therefore, a more positive consumer attitude towards functional foods can be achieved by changing individuals’ perceptions of the importance of diet for health, and by actively demonstrating and publicizing the health benefits of functional foods.

Second, subjective norms had no significant effect on consumption intentions in the context of functional foods (*β* = 0.118, *p* = 0.222, see Table 4) in this study. This finding contradicts theoretical expectations and some prior empirical findings (14, 24). However, this lack of influence is consistent with comprehensive meta-analytic evidence indicating that subjective norms have limited predictive value with regard to food consumption behaviors (41). Patch et al. also demonstrated that subjective norms did not significantly impact Australians’ intentions to consume foods enriched with omega-3 fatty acids (51). Possible reasons for this are that, on the one hand, the decision to consume functional foods is more dependent on the internal factors of the individual consumer, such as their own health priorities, health needs, and level of nutritional knowledge. Consequently, when purchasing functional foods, consumers are more likely to pay attention to objective information, such as the actual efficacy and ingredients of the products, rather than the opinions of others. On the other hand, the regulatory system for functional foods in China is currently not robust and gaps and ambiguous areas in the regulations exist. In particular, the lack of effective regulation of live e-commerce has led to several chaotic phenomena in the current functional food market, such as false propaganda, exaggerated effects, uneven product quality, and other issues, which have led to general scepticism of functional foods (14). This distrust of the market weakens the influence of subjective norms (e.g., peer recommendations, social pressure, etc.) on consumers’ purchase intentions. Consequently, subjective norms did not have a significant effect on purchase intention in this study.

Third, perceived behavioral control is an important predictor of functional food consumption intentions (*β* = 0.148, *p* = 0.002, see Table 4) and behaviors (*β* = 0.841, *p* < 0.001, see Table 4), this aligns with foundational Theory of Planned Behavior (TPB) postulates and corroborates prior empirical work (49, 76). If consumers have a more positive perception of a product in terms of self-efficacy, convenience, and price perception, their consumption behavior will be significantly facilitated. It may be beneficial to increase product availability to increase the consumption of functional foods by Chinese consumers. This is consistent with Marimuthu’s findings on the convenience-driven decision-making of young mothers (54), as well as with Lalor et al.’s emphasis on price sensitivity in the adoption of healthy foods (56). Notably, while PBC has a stronger direct effect on behavior (*β* = 0.841) than on intention (*β* = 0.148), this discrepancy underscores an important insight: consumers may intend to consume functional foods based on attitude, social influence or trust, but they only translate these intentions into action when practical barriers are surmounted.

Fourth, trust shows a significant positive relationship with consumers’ intention to purchase functional foods (*β* = 0.115, *p* < 0.001, see Table 4). This finding is consistent with previous studies (16, 63, 77, 78), which indicate that higher safety and lower risks enhance consumers’ willingness to purchase healthy foods perceived as nutritionally superior to other options. Consumers’ intention to purchase functional foods increases with trust; the key challenge in functional food development is to provide consumers with solid assurances so that they believe in the efficacy and safety of functional foods (79). The current mistrust of Chinese consumers towards functional foods not only hinders the consumption of these foods by individuals, but also limits the healthy development of the functional food industry.

Fifth, our analysis reveals that there is no statistically significant relationship between consumers’ intentions to purchase functional foods and their actual consumption behavior (*β* = 0.002, *p* = 0.973, see Table 4). This finding contradicts previous research on health behaviors (31), revealing a significant discrepancy between intentions and behavior within China’s functional food market. Although consumers may express positive intentions regarding their consumption, the ability to translate these intentions into action appears to be critically dependent on external constraints. A possible reason for this is that consumers may have ideas about consuming functional foods; however, for various reasons, including information, external factors, and complex decision-making, it is difficult to directly translate these ideas into actual consumption behavior. The minimal impact of intention on consumption behavior, combined with the substantial influence of PBC, indicates that strategies that focus solely on enhancing intention are insufficient. To bridge this intention-behavior gap, systematic interventions are required to eliminate environmental barriers.

### 5.2 Practical implications

Based on these findings, several recommendations have been made to improve Chinese consumers’ intentions to consume functional foods and improve their behavior.

In this study, attitude is the strongest factor influencing the intention to consume functional foods, while health consciousness positively influences attitude. Based on this, on the one hand, it is recommended that consumer education be strengthened. Professionals can explain the composition, efficacy, and applicable population of functional foods to correct consumer misconceptions by organizing health lectures and science popularization activities. Popular science articles and videos should be published using new internet-based media to present scientific evidence and latest research results on functional foods and to raise public awareness. There is a need for targeted nutrition education for specific groups, which in turn will improve the knowledge and attitudes of the target groups towards functional foods (55). On the other hand, given that consumers cannot assess the health benefits of a food product solely by its appearance, health claims have become the most important means of direct dialogue between food manufacturers and consumers (80). Therefore, a health labeling campaign is proposed, which would require enterprises to transform scientifically validated health claims into legally binding quality commitment clauses.

This study shows that trust is a key factor influencing Chinese consumers’ purchases of functional foods. Regaining the trust of consumers frustrated by market disruptions is a top priority. To this end, it is crucial to strengthen the supervision of the entire production and distribution chain and rebuild the ecology of market confidence (81). In the production chain, implementing “Good Manufacturing Practice” (GMP) certification requires enterprises to establish a comprehensive traceability system spanning from the procurement of raw materials to the manufacture of finished products. At the distribution stage, supervision over online and offline sales channels must be enhanced and false advertising must be investigated. To address issues in live-streaming platforms, reference can be made to the practice of the Shanghai Municipal Health Commission and the Shanghai Patriotic Health Campaign Committee Office in establishing an internet “Negative Behavior List,” which prohibits the dissemination of false or erroneous health science and technology information and exaggerated claims about disease treatment efficacy (82).

This study indicates that perceived behavioral control influences consumers’ purchase intentions and behavior. Emphasizing that the product is suitable for a specific group of people so that consumers feel that the product can meet their needs and increase their confidence in purchasing it is pivotal to improving consumer self-efficacy and control in the consumption of functional foods. In product packaging and advertising, the composition of the product ingredients, specific effects, correct use of the product, and related precautions should be presented in detail so that consumers can clearly understand the process of using the product and its expected results, thereby reducing uncertainty. In terms of price, it is important to regulate the market price mechanism, curb the phenomenon of inflated prices through market research, and develop a reasonable price range so that consumers can experience the cost-effectiveness of the product and rest assured of the worth of the purchase. In addition, a government subsidy scheme could be implemented for food enterprises or consumers to incentivize the development and launch of affordable functional foods. This approach would enable low-income groups to benefit from functional foods and participate seamlessly in the market, thereby enhancing food security and nutritional equity.

## 6 Limitations and direction for future research

Despite the strengths of this study, such as empirically validating the explanatory power of the Theory of Planned Behavior (TPB) in the context of functional food consumption and expansion of the theoretical framework by integrating novel factors, there remain limitations. The main one being the sampling method. While the sample size was suitable and demographically balanced, collecting the data online excluded less digitally literate and older rural populations, which limited the validity of extrapolating these findings to the entire Chinese functional food consumer population. In future studies, a combination of online and offline surveys can be used to extend the coverage of the population. Second, this study considered the consumption behavior of “functional foods” in general and did not focus on a specific type of functional food, and the results may be different when specific functional foods are considered, i.e., the strength of each pathway in the model. Future research should test this model using a variety of functional foods, including functional dairy products, beverages, and foods that improve blood glucose levels. Third, although the reliability of the scales met the recommended thresholds, AVE and CR values of some constructs were low. A possible reason for this is that China is a large geographical area, and respondents from different cultures may have understood the same question differently. In future studies, we will ensure that the items in the questions are meaningful and differentiated and increase the number of items.

## 7 Conclusion

This study contributes to the existing literature by providing empirical evidence for the ability of an expanded TPB to predict or explain functional food consumption intentions and behaviors in China. Specifically, attitude is the strongest predictor of Chinese consumers’ intention to purchase functional foods. Perceived behavioral control and trust also determine purchase intention, with perceived behavioral control directly influencing functional food consumption behavior. To address misconceptions and enhance consumer awareness, we recommend strengthening educational initiatives. These initiatives should include health seminars, the dissemination of science-based online content, and targeted nutrition programs. These programs should be led collaboratively by healthcare professionals and producers. Meanwhile, food regulatory authorities should improve their supervision of the entire supply chain, from production to distribution. Furthermore, structural interventions are critical to guarantee equitable access for low-income populations. Such interventions may include regulating market pricing mechanisms and implementing government subsidies. Together, these measures could encourage moderate adoption among current non-consumers, providing significant societal benefits. Future policy and industry initiatives should prioritize the removal of real-world constraints to transform latent demand into sustained consumption of functional foods.

## Data Availability

The raw data supporting the conclusions of this article will be made available by the authors, without undue reservation.

## Appendix

**Table A1:** Questionnaire.

Constructs	Items	Measure/Question
Attitude	ATT1	Regular consumption of functional foods would be beneficial.
	ATT2	Regular consumption of functional foods would be pleasant.
	ATT3	Regular consumption of functional foods would be valuable.
	ATT4	Regular consumption of functional foods would be good.
Subjective norms	SN1	My acquaintances believe that I should consume functional foods regularly.
	SN2	My acquaintances expect me to consume functional foods regularly.
	SN3	My acquaintances endorse my regular consumption of functional foods.
	SN4	My acquaintances understand my regular intake of functional foods.
Perceived behavioral control	PBC1	I have complete control over whether I consume functional foods regularly.
	PBC2	My regular consumption of functional foods is beyond my control (reverse scored).
	PBC3	The decision to consume functional foods regularly lies entirely within my control.
Health consciousness	HC1	I perceive myself as health-conscious.
	HC2	I am willing to make significant sacrifices to maintain a healthy diet.
	HC3	I believe that knowledge of healthy eating practices is essential.
Trust	TR1	I completely trust functional foods to meet nutritional needs.
	TR2	I completely trust food safety regulatory authorities.
	TR3	I completely trust in the integrity of functional food producers.
Intention	INT1	I intend to eat functional foods regularly.
	INT2	I expect to eat functional foods regularly.
	INT3	I try to eat functional foods regularly.
	INT4	I plan to eat functional foods regularly.
Behavior	BEH1	How often do you eat functional foods?
	BEH2	How long have you consumed functional foods as part of your daily diet?
	BEH3	When purchasing food, what percentage of your total food consumption consists of functional foods?
